# Unveiling the clinical and genetic impact of neuropsychiatric involvement in systemic lupus erythematosus

**DOI:** 10.1136/rmdopen-2025-006033

**Published:** 2025-10-07

**Authors:** Soojin Cha, Ga Young Ahn, Kwangwoo Kim, Hye-Soon Lee, Sang-Cheol Bae, So-Young Bang

**Affiliations:** 1 Rheumatology Research, Hanyang University Institute for Rheumatology Research, Seoul, Korea (the Republic of); 2 Hanyang Institute of Bioscience and Biotechnology, Seoul, Korea (the Republic of); 3 Rheumatology, Department of Internal Medicine, Korea University Guro Hospital, Seoul, Korea (the Republic of); 4 Department of Biology, Kyung Hee University, Seoul, Korea (the Republic of); 5 Department of Biomedical and Pharmaceutical Sciences, Kyung Hee University, Seoul, Korea (the Republic of); 6 Department of Rheumatology, Hanyang University Hospital for Rheumatic Diseases, Seoul, Korea (the Republic of)

**Keywords:** Lupus Erythematosus, Systemic, Risk Factors, Polymorphism, Genetic

## Abstract

**Objectives:**

Despite the clinical significance of neuropsychiatric systemic lupus erythematosus (NPSLE), which is a severe complication of SLE, clinical and genetic studies on NPSLE remain limited. This study aimed to identify the clinical features of NPSLE and explore genetic factors associated with NPSLE in a prospective lupus cohort.

**Methods:**

The clinical features, disease activity and organ damage of 1205 Korean patients with SLE were assessed at least annually, and genome-wide genotyping with imputation was performed. The clinical and genetic associations of NPSLE, excluding minor events, and its specific subsets (seizure or psychosis) compared with those of non-NPSLE were analysed using genome-wide association studies (GWASs). The biological relevance of the identified loci was investigated through functional analyses.

**Results:**

A total of 271 patients with NPSLE exhibited more clinically diverse manifestations (p=2.40×10^−4^), especially in patients with seizure (N=84, p=4.86×10^−14^) and psychosis (N=26, p=8.29×10^−5^), compared with 934 patients with non-NPSLE. Notably, NPSLE patients significantly had greater organ damage (total Systemic Lupus International Collaborating Clinics/American College of Rheumatology Damage Index (SDI) score: OR 1.49, p=3.28×10^−17^; non-NP SDI score: OR 1.22, p=7.61×10^−5^) than patients with non-NPSLE, adjusting for age, sex, hypertension, disease duration and antiphospholipid antibodies. GWAS revealed nine NPSLE-associated loci at a suggestive significance level (p<5×10⁻⁶). The nine nearest mapped genes were exclusively expressed in the brain (adjusted p=5.28×10^−4^), particularly in the basal ganglia, cortex and hippocampus (adjusted p<0.05).

**Conclusions:**

Neuropsychiatric involvement in SLE increases clinical manifestations and extends organ damage beyond the nervous system, with NPSLE-related genetic variants highlighting their potential functional roles in various brain regions.

**WHAT IS ALREADY KNOWN ON THIS TOPIC**

- Neuropsychiatric systemic lupus erythematosus (NPSLE) is a severe complication of SLE, characterised by a heterogeneous and diverse spectrum of clinical manifestations.
- Clinical and genetic studies focused on major NPSLE subsets, such as seizure or psychosis, remain limited, thereby restricting insights into the pathogenesis of clinically significant neuropsychiatric manifestations.

**WHAT THIS STUDY ADDS**

- In our prospective lupus cohort, NPSLE patients show more diverse SLE clinical manifestations and greater organ damage compared with non-NPSLE patients.
- Patients with seizure or psychosis exhibit multiple organ involvement and distinct neurological system-related genetic features.
- NPSLE-related genes are significantly enriched in the brain, particularly the basal ganglia, cortex and hippocampus.

**HOW THIS STUDY MIGHT AFFECT RESEARCH, PRACTICE OR POLICY**

- These findings enhance understanding of the genetic basis and neurobiological mechanisms of NPSLE and provide new insights into its systemic and neurological burden.
- The results may inform future research on disease pathogenesis and support personalised monitoring and management strategies for SLE patients with neuropsychiatric involvement.

## Introduction

 Neuropsychiatric (NP) involvement in systemic lupus erythematosus (SLE), representing a heterogeneous and diverse spectrum of manifestations, is collectively referred to as neuropsychiatric systemic lupus erythematosus (NPSLE). It is characterised by 19 distinct features as defined by the 1999 NP American College of Rheumatology (ACR) criteria.^1^ NPSLE has a significant source of morbidity and exhibits mortality rates second to those of lupus nephritis.^2^ Recent advances in understanding the genetics of SLE through genome-wide association studies (GWASs) have identified more than 180 SLE-related genetic loci, providing critical insights into its genetic basis.^3^,^5^ These large-scale studies have also highlighted the role of genetic contributions to organ damage, underscoring the complexity of SLE pathogenesis. Nevertheless, despite the clinical severity of NPSLE, the genetic loci specifically associated with major NP manifestations remain undefined.

Seizure and psychosis are well-recognised major NP events associated with severe disease, and persistently positive antiphospholipid antibodies are associated with an increased risk of NPSLE, particularly in patients with seizure.^6^ However, the genetic and clinical risk factors of SLE patients with major NP events remain unclear, thus posing a major challenge to understanding its pathogenesis. Moreover, although both are classified as NPSLE, seizure and psychosis may represent distinct pathophysiological entities.

One of the greatest challenges in studying NPSLE is its heterogeneity. Since many studies have not distinguished between mild NP symptoms and severe, clinically significant manifestations such as seizure and psychosis, the prevalence of NPSLE is estimated to range from 20% to 70%.^7^ Despite its heterogeneity, previous genetic studies analysed NPSLE as a single entity without considering potential differences in risk predisposition across different NP subtypes.^7^,^9^ Previous studies also implicated several genetic factors in NPSLE. For instance, *TREX1* mutations are linked to the increased type I interferon expression and seizure susceptibility in SLE, and *STAT4* polymorphisms are associated with stroke in patients with SLE, independent of the antiphospholipid antibody status.^10^,^12^ However, these studies have primarily focused on specific candidate SLE-associated genes rather than genome-wide analyses and have grouped all NP symptoms, potentially including nonspecific or mild events with uncertain relevance to NPSLE. Given that genetic factors related to SLE and those linked to NP manifestations can substantially differ, GWASs are crucial. These analyses should be performed to explore distinct clinical subsets and major clinical NP manifestations comprehensively.

In this study, we examined NPSLE-related genetic and clinical factors in a prospective Korean lupus cohort. We excluded mild symptoms that are common but not typically associated with SLE, such as headaches, anxiety, mild depression, mild cognitive dysfunction and polyneuropathy without electrophysiological confirmation as defined by Ainiala *et al*.^13^ We conducted GWAS to identify NPSLE-associated genetic loci, comparing to non-NPSLE patients. Given the heterogeneity of NPSLE, we also focused on seizure and psychosis which represent the most severe and clinically relevant NP manifestations of SLE.

## Methods

### Clinical characteristics of patients with SLE

A total of 1205 Korean patients with SLE were enrolled from a prospective BAE lupus cohort^14^ at Hanyang University Hospital. All of them had been under follow-up for more than at least 1 year. NPSLE was defined annually based on 1999 ACR nomenclature and case definitions for NP lupus syndromes, which provide detailed recommendations for diagnostic workup, laboratory and imaging tests and differential diagnosis. We then reapplied a modified version defined by Ainiala in 2001; it consists of 19 ACR criteria but excludes minor NP events.^1^ These exclusions are conditions such as headaches, anxiety disorders, mild cognitive complaints, mild depression and polyneuropathy, unless it is confirmed by electrophysiological evidence.^13^ Seizure and psychosis were analysed as independent manifestations, and also in combination, reflecting their inclusion as key NP symptoms of SLE in both the 1997 revised ACR criteria^15^ and the 2012 Systemic Lupus International Collaborating Clinics (SLICC) criteria^16^ for SLE classification; therefore, they were analysed separately and collectively in this study. Patients with SLE were classified into two groups: those with major NP manifestations based on the Ainiala criteria (NPSLE)^13^ and those without NPSLE manifestations (non-NPSLE). All clinical data were collected and reviewed by experienced rheumatologists. Among patients with NPSLE, seizure and psychosis were analysed separately (online supplemental figure 1).

Clinical features, including disease activity and organ damage, were collected in all patients with SLE. Disease activity was determined using the SLE Disease Activity Index 2000 (SLEDAI-2K). The adjusted mean SLEDAI-2K was calculated by determining the area under the curve of SLEDAI-2K over time, summing the areas of each visit interval and dividing by the follow-up period. NP characteristics, including seizure, psychosis, organic brain syndrome, cranial nerve disorder, visual disturbance, cerebrovascular accident and lupus headache, were excluded to calculate non-NP SLEDAI-2K. Organ damage was assessed using the SLICC Damage Index/ACR Damage Index (SDI) score. The total SDI score included 41 items, while the score of SDI excluding NP items (non-NP) was calculated excluding NP items such as seizures, psychosis, cognitive dysfunction, strokes and neuropathy. This study is reported in accordance with the Strengthening the Reporting of Observational Studies in Epidemiology guidelines.^17^

### Statistical analysis for clinical variables

Wilcoxon rank sum test for continuous variables and χ^2^ test for categorical variables were performed to compare clinical variables between the patients with NPSLE (or seizure, psychosis) and non-NPSLE. Clinical manifestations were defined on the basis of 10 of 11 clinical items (excluding NP manifestations) and three autoantibodies (anti-Sm, anti-dsDNA and antiphospholipid antibodies) from the 1997 ACR criteria for SLE. All patients were ANA positive (titres≥1:80). The significance threshold was set using the Bonferroni method, adjusting for 13 clinical manifestations (p<0.004 (=0.05/13)). Multiple testing correction was applied when comparing clinical manifestations. Multivariable logistic regression analysis was conducted, adjusting for age, sex, hypertension and disease duration, to assess the differences in organ damage and disease activity between NPSLE and non-NPSLE.

### Genotyping, quality control, imputation and genomic analyses

The following NPSLE subsets were analysed to identify NPSLE-associated loci: NPSLE, seizure and/or psychosis (online supplemental figure 2). All patients with SLE were genotyped using a customised genome-wide single nucleotide polymorphism (SNP) array, namely, the Korea Biobank Array (Korean Chip, 833K variants).^18^ After quality control (QC), as described in our previous study,^19^ genotype imputation was conducted by using Eagle V.2.4, Minimac4 and the 1000 Genomes Project Phase 3 V.5 imputation reference panel.^20^ A GWAS was performed on allelic dosage to identify NPSLE-associated genetic loci. Genetic principal component (PC) analysis was conducted to determine population stratification and computed for each individual by using pruned variants (--indep-pairwise 50 5 0.5) with a minor allele frequency of ≥5% and a genotyping rate of at least 98%, as implemented in PLINK 1.9.^21^ For multivariable analysis, logistic regression analysis with the Wald test implemented in R V.4.3.1 was used.

GWASs were conducted for each subset by using a generalised linear mixed model in SAIGE V.1.1.6, adjusting for age, sex and 10 genetic PCs.^22^ Within the FUMA platform V.1.5.2, independent significant SNPs were defined as those with r^2^<0.6 and p<5×10^−6^; lead SNPs were defined as the most significant SNPs among them. Variants that passed post-QC were considered the final loci in each set, and the genomic inflation factor (λ_GC_) was assessed for the NPSLE, seizure or psychosis sets (online supplemental figure 3).

### Functional analyses

Functional analyses were performed to explore the functional roles of the loci identified in NPSLE. SNPs in linkage disequilibrium (LD) with the lead SNP (R^2^>0.6) were assessed to determine whether they were reported as expression quantitative trait loci (eQTL) in the PsychENCODE, GTEx V.8 and BRAINEAC within the FUMA platform.^23 24^ eQTL data from PsychENCODE, BRAINEAC and GTEx V.8 were obtained from their respective databases.^23^,^25^ The normalised effect size and the m-value that represent the posterior probability of the variant’s effect on a tissue (range, 0–1, where 0 indicates no effect, and 1 indicates certainty) were calculated using METASOFT in our multitissue eQTL analysis via GTEx to determine whether an eQTL-gene pair was significantly more effective in specific tissues than in multiple tissues.^26 27^ For variant-gene mapping, ANNOVAR or the nearest gene described in Open Targets Genetics was used.^28^ The genes mapped by ANNOVAR or the nearest genes were used in gene set enrichment analyses (hypergeometric test) against predefined gene sets in the GWAS catalogue and GTEx V.8 within the FUMA platform.

## Results

### Clinical features of patients with NPSLE

Of the 1205 patients with SLE, 22.5% (N=271) had NPSLE, as defined by the modified version of the ACR case definitions for 19 NP syndromes excluding minor NP events based on the Ainiala criteria.^13^ The clinical characteristics of the patients with NPSLE and their specific subsets (seizure (N=84) and/or psychosis (N=26)) and the patients with non-NPSLE (N=934) are presented in table 1. Sex and onset age did not differ between the patients with NPSLE (93.0% female, 26.2 years) and non-NPSLE (91.6% female, 27.0 years). The onset age of the patients having NPSLE with seizures (N=84) was significantly younger (21.9 years, p=3.89×10^−6^) than that of the patients with non-NPSLE. In our SLE cohort, 26 (2.16%) patients exhibited psychosis, with a mean onset age of 24.5 years and a predominance of females (96.2%). The prevalence of hypertension in the patients with NPSLE was higher than that of the patients with non-NPSLE (19.9% vs 13.9%, p=0.02). The mean disease durations of the patients with non-NPSLE and NPSLE were 14.4 and 15.6 years, respectively. Disease duration and follow-up duration did not significantly differ between the two groups.

**Table 1:** Clinical characteristics of NPSLE compared with non-NPSLE in the SLE cohort (N=1205)

	Non-NPSLE* (N=934)	NPSLE (N=271)					
		Total (N=271)	P value†	Seizure (N=84)	P value†	Psychosis (N=26)	P value†
Female (%)	91.6	93.0	0.56	88.1	0.36	96.2	0.64
Age (mean±SD), years	41.4±12.4	41.8±12.7	0.80	36.3±11.3	**1.58×10^-4^**	38.1±9.6	0.15
Onset age (mean±SD)	27.0±10.4	26.2±10.9	0.25	21.9±10.3	**3.89×10^-6^**	24.5±8.9	0.38
Disease duration (mean±SD)	14.4±7.1	15.6±7.3	**0.013**	14.4±7.2	0.87	13.5±7.2	0.63
BMI (kg/m²; mean±SD)	21.3±3.1	21.4±3.1	0.50	21.4±3.3	0.79	21.5±2.6	0.60
Marriage (%)	47.0	49.8	0.43	35.7	0.052	46.2	1.00
Smoker (%)	15.5	14.0	0.67	9.5	0.19	3.8	0.17
Hypertension (%)	13.9	19.9	**0.020**	16.7	0.60	15.4	1.00
Malignancy (%)	1.8	3.3	0.21	3.6	0.49	0	1.00
Diabetes mellitus (%)	4.7	7.0	0.18	6.0	0.81	0	1.00
Clinical manifestations‡‡							
Number of ACR (mean±SD)	5.8±1.4	6.2±1.6	**2.40×10^-4^**	7.2±1.5	**4.86×10^-14^**	7.1±1.7	**8.29×10^-5^**
Malar rash (%)	46.6	49.4	0.45	59.5	0.03	57.7	0.36
Discoid rash (%)	10.4	7.7	0.24	10.7	1.00	11.5	1.00
Photosensitivity (%)	35.9	33.6	0.60	36.9	0.89	42.3	0.65
Oral ulcers (%)	40.3	44.3	0.28	46.4	0.34	57.7	0.12
Arthritis (%)	66.2	58.7	0.03	53.6	0.03	53.8	0.27
Serositis (%)	34.6	45.4	**0.002**	52.4	**0.002**	42.3	0.55
Renal disorder (%)	57.8	60.1	0.51	67.9	0.07	65.4	0.57
Haematological disorder (%)	93.9	92.6	0.46	92.9	0.83	88.5	0.44
Immunological disorder (%)	93.1	93.4	0.90	94.0	0.65	92.3	1.00
Antiphospholipid antibodies	38.5	44.3	0.097	56.0	**0.002**	15.4	0.03
Anti-Sm	33.4	31.7	0.65	35.7	0.76	34.6	1.00
Anti-dsDNA	86.6	86.3	0.96	85.7	0.93	92.3	0.59
ANA (%)	100	100	1.00	100	1.00	100	1.00

* Mean±SD and count (percentage, %) were used for continuous and categorical variables, respectively.

† P values were estimated by comparing individuals between NPSLE (total, seizure or psychosis) and non-NPSLE by using Wilcoxon rank sum test (continuous variables) and χ2 test (categorical variables). Significant p values were in bold.

‡ Clinical manifestations were defined on the basis of 10 of 11 clinical items (excluding NP) from the 1997 ACR criteria and 3 autoantibodies for SLE. The significance threshold was determined using the Bonferroni method, adjusting for the number of clinical manifestations (p<0.004 (=0.05/13)).

ACR, American College of Rheumatology; BMI, body mass index; NP, neuropsychiatric; SLE, systemic lupus erythematosus.

The diverse clinical manifestations of SLE were analysed as the number of ACR criteria based on 11 items from the 1997 ACR classification criteria.^15^ The results revealed that the number of clinical manifestations of the patients with NPSLE was significantly more than those of the patients with non-NPSLE (number of ACR, 6.2 vs 5.8, p=2.40×10^−4^, table 1). Moreover, the clinical manifestations of SLE in patients with seizure or psychosis were more diverse (number of ACR, 7.2 vs 5.8, p=4.86×10^−14^ and 7.1 vs 5.8, p=8.29×10^−5^, respectively). Serositis was more frequently observed in patients with NPSLE (p=0.002), and its prevalence was particularly higher in patients having seizures (p=0.002, adjusted threshold of p<0.004). Additionally, the prevalence of antiphospholipid antibodies in patients with seizures was higher than that in patients with non-NPSLE (56.0% vs 38.5%, p=0.002; table 1).

### Organ damage and disease activity of patients with NPSLE

Organ damage was annually assessed in terms of the SDI score of all patients with SLE. Notably, it was significantly greater in patients with NPSLE than in patients with non-NPSLE, as indicated by the total SDI and the non-NP SDI scores (OR 1.49, p=3.28×10^−17^; OR 1.22, p=7.61×10^−5^, respectively), after adjustment for age, sex, hypertension, disease duration and antiphospholipid antibodies (table 2). Specifically, the SDI scores of patients with seizures (total SDI (OR 1.77, p=4.91×10⁻^16^, non-NP SDI (OR 1.35, p=5.55×10⁻⁵)) and psychosis (total SDI (OR 1.70, p=1.08×10⁻⁵) and non-NP SDI (OR 1.32, p=0.045)) were higher than those of patients with non-NPSLE. Analysis by SDI domain revealed a significantly higher frequency of musculoskeletal damage in the NPSLE group (OR 1.81, p=2.65×10⁻⁴) (online supplemental figure 4). Within NPSLE subgroups, this association was present in the seizure subgroup (OR 2.13, p=4.22×10⁻³), whereas the psychosis subgroup showed no significant differences compared with non-NPSLE. Therefore, the damage experienced by patients with NPSLE was likely more severe than that suffered by patients with non-NPSLE.

**Table 2:** Organ damage and disease activity in patients with NPSLE

	NPSLE (N=271)		Seizure (N=84)		Psychosis (N=26)	
	OR (95% CI)*	P value*	OR (95% CI)*	P value*	OR (95% CI)*	P value*
Organ damage†						
SDI (total)	1.49 (1.36 to 1.63)	**3.28×10^–17^**	1.77 (1.54 to 2.04)	**4.91×10^−16^**	1.70 (1.34 to 2.15)	**1.08×10^−5^**
SDI (non-NP)	1.22 (1.1 to 1.34)	**7.61×10^–5^**	1.35 (1.17 to 1.56)	**5.55×10^−5^**	1.32 (1.01 to 1.74)	**0.045**
Disease activity‡						
Adjusted mean SLEDAI-2K, non-NP§	1.02 (0.96 to 1.09)	0.52	1.12 (1.02 to 1.23)	**0.019**	1.17 (1.00 to 1.37)	0.057
SLEDAI-2K, non-NP (enrol)	0.98 (0.95 to 1.01)	0.23	1.00 (0.94 to 1.05)	0.89	1.00 (0.91 to 1.09)	0.97
SLEDAI-2K, non-NP (last)	1.02 (0.98 to 1.05)	0.39	1.06 (1.00 to 1.11)	0.05	1.06 (0.96 to 1.16)	0.26

* ORs and p values for NPSLE were calculated and compared with non-NPSLE after adjustments for age, sex, hypertension, disease duration and antiphospholipid antibodies. Significant P values (< 0.05) are highlighted in bold.

† Organ damage was assessed using the SDI score. The SDI (non-NP) score, excluding NP items such as seizures, psychosis, cognitive dysfunction, strokes and neuropathy, was used to assess organ damage.

‡ Disease activity was assessed using the SLEDAI-2K at enrolment (enrol) and annually until the last follow-up visit (last). The adjusted mean SLEDAI-2K was determined through the calculation of the area under the curve of SLEDAI-2K over time by adding the area of each of the visit intervals and dividing by the follow-up period.

§ Non-NP SLEDAI-2K were the excluded neuropsychiatric characteristics, such as seizure, psychosis, organic brain syndrome, cranial nerve disorder, cerebrovascular accident and lupus headache.

ACR, American College of Rheumatology; NP, neuropsychiatric; SDI, SLICC/ACR Damage Index; SLE, systemic lupus erythematosus; SLEDAI-2K, SLE Disease Activity Index 2000; SLICC, Systemic Lupus International Collaborating Clinics.

Disease activity was assessed using the SLEDAI-2K at enrollment and at least once annually until the last follow-up visit. The disease activity, excluding NP, of the patients with seizure during the follow-up period was higher than that of the patients with non-NPSLE (adjusted mean SLEDAI-2K OR 1.12, p=0.019), adjusted for age, sex, hypertension, disease duration and antiphospholipid antibodies (table 2). When we compared each component of adjusted mean SLEDAI-2K, thrombocytopenia was significantly more observed in the seizure subgroup compared with non-NPSLE groups (p=0.001).

### GWAS and functional analyses of NPSLE

To identify NPSLE-associated genetic loci, we conducted GWAS across specific NPSLE subsets (NPSLE, seizure or psychosis) and compared them with non-NPSLE by using a generalised linear mixed model within SAIGE, adjusting for age, sex and 10 genetic PCs^22^ described in our workflow (online supplemental figure 2). We identified nine NPSLE-associated loci at a suggestive significance level (p<5×10^−6^; table 3, online supplemental table 1 and online supplemental figure 5).

**Table 3:** Nine NPSLE-associated loci* compared with non-NPSLE

Lead SNP	Chromosome: Base position (GRCh37)	Effect allele	P value	Allele frequency		Nearest genes
				NPSLE	Non-NPSLE	
rs62838161	12:117 782 836	C	3.75×10^-7^	0.709	0.801	*NOS1*
rs201681644	8:18 401 681	C	6.58×10^-7^	0.959	0.991	*PSD3*
rs4508395	15:78 239 225	T	6.82×10^-7^	0.211	0.131	*LINGO1*
rs75831230	10:61 972 855	A	1.39×10^-6^	0.037	0.011	*ANK3*
rs6023524	20:53 310 831	C	1.48×10^-6^	0.224	0.329	*DOK5*
rs56405523	11:133 377 750	C	2.20×10^-6^	0.294	0.207	*SLC24A4*
rs16984880	2:18 578 361	A	3.33×10^-6^	0.079	0.034	*RDH14*
rs34875253	14:92 959 361	G	3.72×10^-6^	0.294	0.207	*SLC24A4*
rs185044622	8:9 292 316	T	4.99×10^-6^	0.043	0.013	*TNKS*

* NPSLE-associated loci at the suggestive significance level (<5×10−6).

NPSLE, neuropsychiatric systemic lupus erythematosus; SNP, single nucleotide polymorphism.

**Figure 1:**
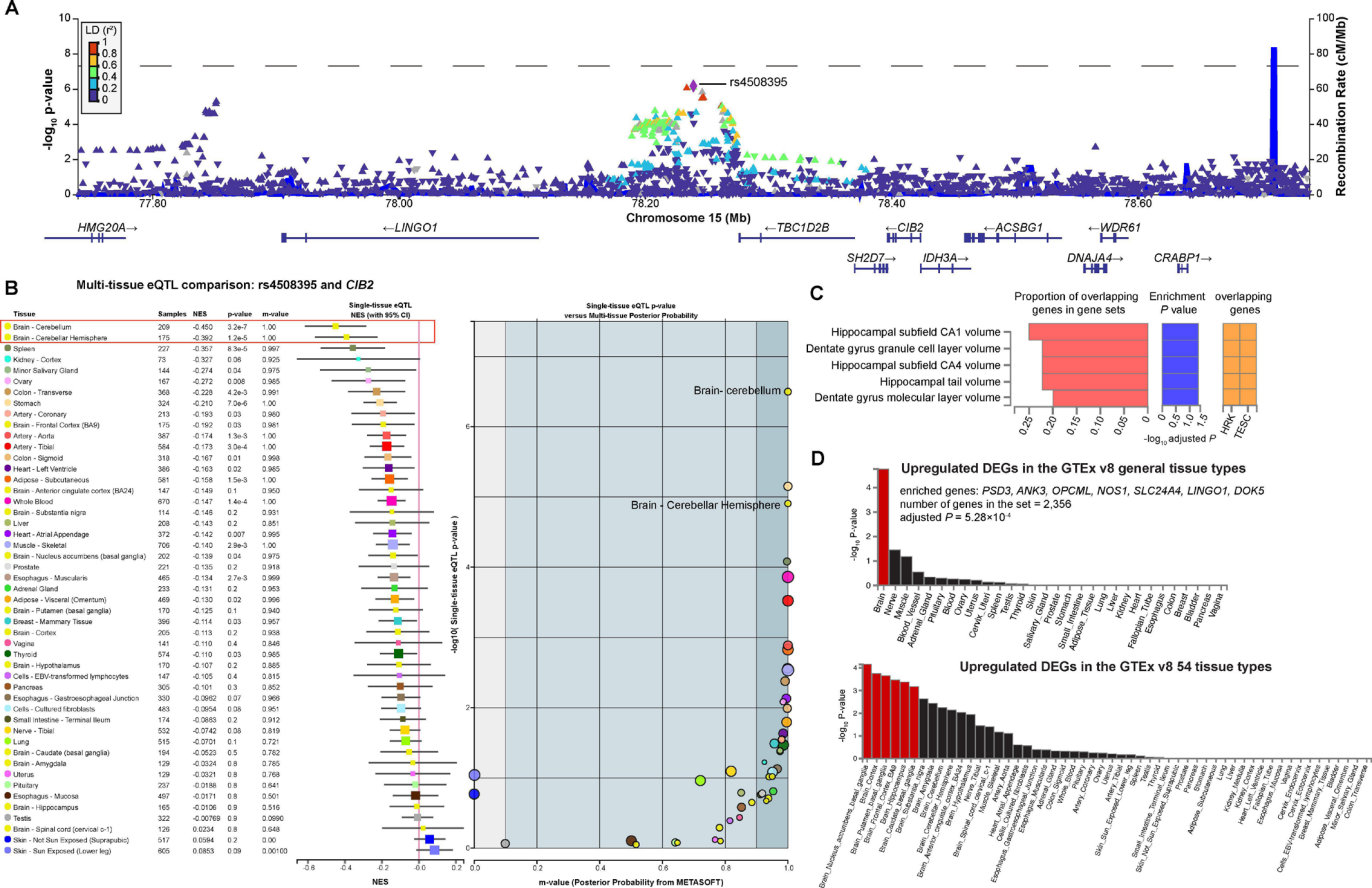
NPSLE-associated loci and their functional implications in the brain. (A) The brain-related locus rs4508395 (15:78239225:C>T) was presented in the regional plot. The lead SNP (rs4508395) was shown in purple, and other SNPs were coloured on the basis of their linkage disequilibrium (LD) with the lead SNP. (B) rs4508395 elicited a cis-eQTL effect on *CIB2* in brain regions, including the cerebellum and cerebellar hemisphere, compared with that on multiple tissue types (m-value >0.9 obtained by METASOFT). (C) Among the 25 mapped genes with NPSLE-associated loci, *HRK* and *TESC* exhibited significant enrichment in gene sets associated with the traits of the hippocampus and dentate gyrus. (D) The nine nearest genes of NPSLE-associated loci were significantly enriched in the brain-related gene sets from the GTEx database. eQTL, expression quantitative trait loci; NPSLE, neuropsychiatric systemic lupus erythematosus; SNP, single nucleotide polymorphism; DEGs; differential expressed genes.

We conducted functional analyses to explore the roles of the loci identified in NPSLE. Specifically, rs4508395 (15:78239225:C>T, p=6.82×10^−7^, figure 1A) showed significant *cis*-eQTL effects on *CIB2* and *DNAJA4* in various brain regions based on a multi-tissue eQTL comparison (figure 1B). It was significantly associated not only with the *CIB2* expression in the cerebellum (p=3.2×10^−7^) and cerebellar hemisphere (p=1.2×10^−5^) but also with the *DNAJA4* expression in the cerebellum (p=1.3×10^−5^) and cerebellar hemisphere (p=7.1×10^−4^; figure 1B and online supplemental figure 6).

Nine significant NPSLE-related loci (p<5×10^−6^) were mapped to 25 genes by using the FUMA platform (online supplemental table 2). Two genes, namely, *HRK* and *TESC*, were significantly enriched in the gene set related to the hippocampal volume according to the GWAS catalogue (adjusted p=0.048, figure 1C). Intriguingly, the nine nearest genes identified from the loci were exclusively enriched in the gene sets highly expressed in the brain (adjusted p=5.28×10^−4^), specifically in the basal ganglia (nucleus accumbens, putamen, caudate), cortex and hippocampus compared with those in multiple other tissues (adjusted p*<*0.05; figure 1D and online supplemental table 3). The nucleus accumbens of the basal ganglia showed the most significant upregulation among the brain regions (p=6.93×10^−5^, adjusted p=3.74×10^−3^, online supplemental table 3). Therefore, NPSLE-associated loci were potentially related to brain-specific tissues.

**Figure 2:**
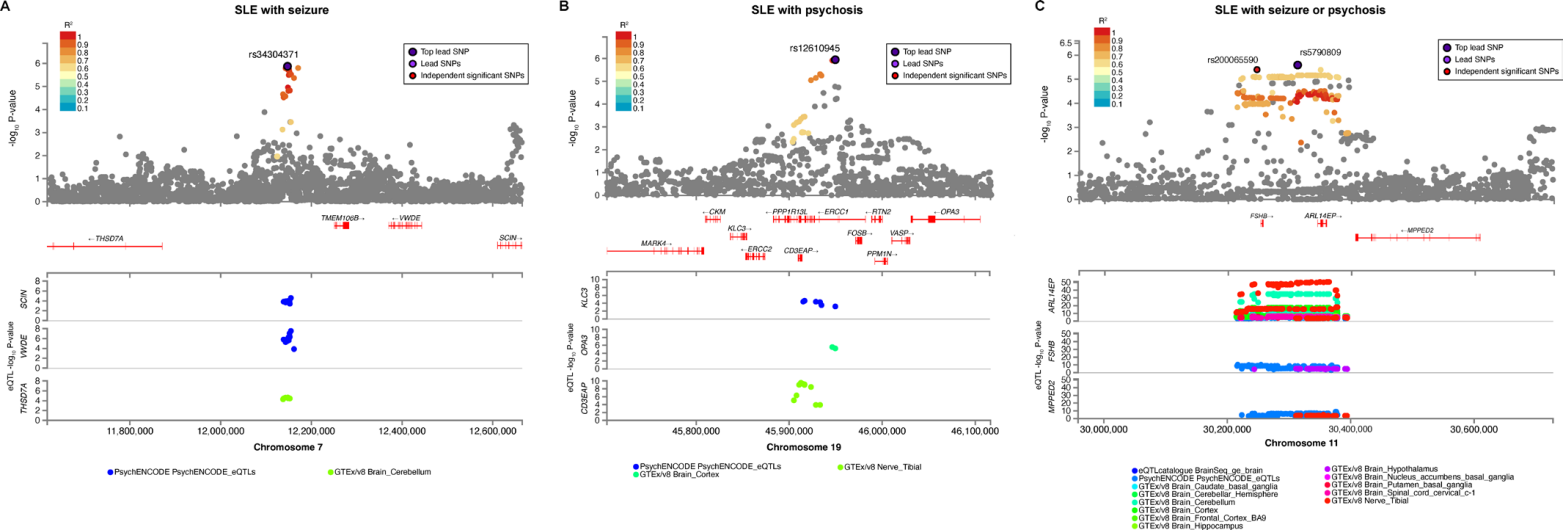
Loci significantly associated with seizure or psychosis highlight the regulation of gene expression in brain regions. (A) SLE with seizure; (B) SLE with psychosis and (C) significant loci associated with SLE with seizure or psychosis. eQTL, expression quantitative trait loci; SLE, systemic lupus erythematosus; SNP, single nucleotide polymorphism.

We analysed seizure and psychosis, recognised as major NP events, using GWAS and functional analyses. We identified seven loci that were associated with seizure at a suggestive significance level of p<5×10^−6^ (online supplemental table 1). rs34304371 (7:12147500: G>A, p=1.32×10^−6^) was the eQTL of *THSD7A*, *VWDE* and *SCIN* in the cerebellum of the brain (figure 2A). Nine loci were related to psychosis (p<5×10^−6^, online supplemental table 1). rs12610945 (19:45949578:C>T, p=1.13×10^−6^) elicited eQTL effects on brain cortex and nerve (figure 2B).

In patients with neurological involvement (seizure or psychosis, N=103) according to the 1997 ACR criteria for SLE, nine loci were identified (online supplemental table 1). rs5790809 (11:30312421, p=2.57×10^-6^) showed numerous eQTL associations in diverse neurological regions (basal ganglia, cerebellum, cortex, hippocampus, hypothalamus, nerve and spinal cord cervical c-1; figure 2C and online supplemental table 4). Most eQTLs were paired with the ADP ribosylation factor-like GTPase 14 effector protein (*ARL14EP*), which participates in axonal development and its expression in neural progenitors.

## Discussion

In this study, we identified the clinical and genetic contributions of NP SLE in a prospective Korean lupus cohort. The patients with NPSLE, specifically excluding minor NP events, exhibited significantly more diverse clinical manifestations and greater organ damage than the patients with non-NPSLE even after adjustment for age, sex, hypertension and disease duration. We comprehensively assessed the disease activity at each visit and organ damage at least annually in a well-organised prospective cohort and revealed novel findings that have not yet been reported in other studies.^29 30^ The clinical manifestations of SLE in patients with seizure or psychosis, which are two severe NP events, were significantly more diverse. Compared with patients with non-NPSLE, patients with seizures had a 2.04-fold increase in total organ damage and a 1.48-fold increase in non-NP organ damage. Patients with psychosis showed a 1.75-fold and 1.42-fold increase, respectively. These findings highlighted the significant systemic burden and severity of these NP manifestations in SLE. Consistent with a previous study,^31^ the present study revealed that seizure was associated with an earlier disease onset and a higher prevalence of antiphospholipid antibodies. Even when NP manifestations were excluded, patients with seizures had significantly higher SLE disease activity compared with those with non-NPSLE.

Interestingly, we demonstrated that the genes associated with NPSLE-associated loci by GWAS were significantly expressed in brain regions, including the basal ganglia, cortex and hippocampus. The loci related to seizure or psychosis were linked to gene regulation in multiple brain regions and neurological systems, thereby highlighting their relevance to NP involvement in SLE.

NPSLE is a major clinical manifestation of SLE, and our findings reinforced the notion that NP involvement in SLE represents a distinct and clinically significant disease subset. However, unlike lupus nephritis, which has clear diagnostic methods such as renal biopsy and objective biomarkers, NPSLE is characterised by its diverse phenotypes, broad spectrum of severity and varying degrees of SLE attribution.^32^ We found few overlapped genetic loci between four analysis sets; as such, patients with NPSLE showed highly heterogeneous characteristics as expected.

With previous findings, our results suggested a possible relevance to a NP function. In the NPSLE set, hippocampal volume related to genes, such as *HRK* and *TESC*, was linked to NPSLE (figure 1C). Previous studies showed that the hippocampus is related to the CNS NPSLE, including cognitive disorders; anti-dsDNA antibodies can interact with NMDA receptors to induce apoptosis in hippocampal neurons.^33^,^35^ Further studies are needed to clarify the pathogenic relevance of these genes to SLE-specific autoimmune mechanisms, particularly in NPSLE. In our analysis, rs62838161, an intronic SNP of neuronal nitric oxide synthase (*NOS1*), was associated with NPSLE. A Japanese study demonstrated that *NOS1* is highly expressed in the brain, and one functional polymorphism in *NOS1* (exon 29) is significantly associated with the schizophrenia risk (p=7×10⁻⁶).^36^

Our psychosis subset analysis revealed that rs139418206, an intronic SNP of *SORCS3,* encodes a neuronal receptor that regulates synaptic remodelling, cognitive stability and emotional regulation. Considering the pleiotropic associations of *SORCS3* variants with NP disorders, including schizophrenia,^37^ rs139418206 may participate in SLE with psychiatry.

We identified rs5790809 from SLE with neurological involvement (seizure or psychosis set), demonstrating its regulatory effects on *ARL14EP*, a gene previously associated with the schizophrenia risk.^38 39^ This regulatory effect was significantly observed in multiple brain regions, including the hippocampus, cortex, basal ganglia and cervical spine, reinforcing its relevance to NP dysfunction. Notably, eQTL effects on the cervical spinal cord have not been reported in patients with NPSLE. Among the LD SNPs of rs200065590, rs1765142 (p=2.00×10^−6^) and rs515997 (p=3.00×10^−9^) were previously linked to schizophrenia (figure 2C).^40 41^

The basal ganglia, cortex and hippocampus, which are the regions affected by NPSLE-associated genes identified in our GWAS, are key brain areas involved in emotion, cognition and memory; their dysfunction has been implicated in the NP pathogenesis of SLE.

Despite the interesting findings, our study has limitations. We applied the Ainiala criteria for the definition of NPSLE, which enhanced diagnostic specificity but may have led to the underrepresentation of mild NP manifestations. Although our study was based on a well-designed, prospective Korean cohort (N=1205), genome-wide significance could not be achieved due to the limited sample size and lack of replication. The identified genes may be relevant to NP manifestations in SLE, but their pathogenic roles remain unclear, and their relevance to NP disorders beyond SLE has not yet been investigated.

Analyses of specific NPSLE-associated autoantibodies (eg, anti-ribosomal P, anti-NR2) and polygenic risk scores were not performed. Future studies in larger multiethnic cohorts with comprehensive autoantibody profiles and polygenic risk assessment are needed to validate our findings and to better elucidate the clinical and genetic heterogeneity of NPSLE. In conclusion, our study characterised the clinical and genetic landscape of NPSLE in a prospective Korean lupus cohort and emphasised its distinct and clinically significant phenotype beyond NP symptoms alone. Patients with NPSLE exhibited more extensive systemic involvement and significantly greater organ damage; thus, NP manifestations contributed to multiorgan effects in SLE. We identified the NPSLE-related genetic loci that showed potential biological functions across multiple brain regions, particularly in the basal ganglia, cortex and hippocampus; such functions suggested that they could be involved in NP pathogenesis. Therefore, these findings may provide exploratory insights into key mechanisms in NPSLE and could contribute to predicting clinical outcomes and advancing the personalised clinical management of SLE.

## Supplementary material

10.1136/rmdopen-2025-006033 online supplemental file 1

## Data Availability

Data are available on reasonable request. All data relevant to the study are included in the article or uploaded as supplementary information.
