# Supplementary material for: Unveiling the clinical and genetic impact of neuropsychiatric involvement in systemic lupus erythematosus
Source: RMD Open. 2025 Oct 7;11(4):e006033. doi: 10.1136/rmdopen-2025-006033 (PMC12506454; doi:10.1136/rmdopen-2025-006033)
Supplement: online supplemental file 1 [file rmdopen-11-4-s001.docx]

**Supplementary material**

**Unveiling the clinical and genetic impact of neuropsychiatric involvement in systemic lupus erythematosus**

Soojin Cha^1,2*^, Ga Young Ahn^3*^, Kwangwoo Kim^4,5^, Hye-Soon Lee^1,2,6^, Sang-Cheol Bae^1,2,6†^, So-Young Bang^1,2,6†^

*^1^ Rheumatology Research, Hanyang University Institute for Rheumatology Research, Seoul, Korea*

*^2^ Hanyang Institute of Bioscience and Biotechnology, Seoul, Republic of Korea
^3^ Rheumatology, Department of Internal Medicine, Korea University Guro Hospital, Korea*

*^4^ Department of Biology, Kyung Hee University, Seoul, Republic of Korea*

*^5^ Department of Biomedical and Pharmaceutical Sciences, Kyung Hee University, Seoul, Republic of Korea*

*^6^ Department of Rheumatology, Hanyang University Hospital for Rheumatic Diseases, Korea*

^*^These authors contributed equally to this paper.

^†^ **Correspondence to** So-Young Bang, Department of Rheumatology, Hanyang University Hospital for Rheumatic Diseases, Seoul, Republic of Korea; sybang@hanyang.ac.kr, or Sang-Cheol Bae, Department of Rheumatology, Hanyang University Hospital for Rheumatic Diseases, Seoul, Republic of Korea; scbae@hanyang.ac.kr

**Supplementary table 1. NPSLE-associated loci at a suggestive significance level (<5×10^-6^) compared to non-NPSLE**

|  | Lead SNP ^*^ | Independent SNPs | Chr:Position  (GRCh37) | Risk allele | Beta | SE | P | Allele frequency | | Nearest genes ^†^ | Annotation ^†^ |
| --- | --- | --- | --- | --- | --- | --- | --- | --- | --- | --- | --- |
|  |  |  |  |  |  |  |  | **NPSLE** | **non-NPSLE** |  |  |
| NPSLE  (N=271) | rs16984880 | rs16984880 | 2:18578361 | A | 1.23 | 0.26 | 3.33×10^-6^ | 0.079 | 0.034 | *RDH14* | intergenic |
|  | rs185044622 | rs185044622 | 8:9292316 | T | 1.86 | 0.41 | 4.99×10^-6^ | 0.043 | 0.013 | *TNKS* | intergenic |
|  | rs201681644 | rs201681644 | 8:18401681 | C | -2.09 | 0.42 | 6.58×10^-7^ | 0.959 | 0.991 | *PSD3* | intronic |
|  | rs75831230 | rs75831230 | 10:61972855 | A | 2.43 | 0.50 | 1.39×10^-6^ | 0.037 | 0.011 | *ANK3* | intronic |
|  | rs56405523 | rs56405523 | 11:133376450 | C | 0.55 | 0.12 | 2.82×10^-6^ | 0.340 | 0.248 | *OPCML* | intronic |
|  | rs62838161 | rs62838161; rs551340 | 12:117782836 | C | -0.67 | 0.13 | 3.75×10^-7^ | 0.709 | 0.801 | *NOS1* | intronic |
|  | rs34875253 | rs34875253 | 14:92959361 | G | 0.59 | 0.13 | 3.72×10^-6^ | 0.294 | 0.207 | *SLC24A4* | intronic |
|  | **rs4508395** | rs4508395 | 15:78239225 | T | 0.74 | 0.15 | 6.82×10^-7^ | 0.211 | 0.131 | *LINGO1* | intergenic |
|  | **rs6023524** | rs6023524 | 20:53310831 | C | -0.53 | 0.11 | 1.48×10^-6^ | 0.224 | 0.329 | *DOK5* | intergenic |
| Seizure  (N=84) | rs16845943 | rs16845943 | 4:72026793 | A | 4.79 | 1.00 | 1.47×10^-6^ | 0.060 | 0.008 | *SLC4A4* | intergenic |
|  | **rs191085932** | rs191085932 | 4:77499261 | C | 3.50 | 0.72 | 1.01×10^-6^ | 0.077 | 0.011 | *SHROOM3* | intronic |
|  | rs80182806 | rs80182806 | 4:179428121 | G | 0.87 | 0.19 | 3.01×10^-6^ | 0.450 | 0.286 | *AGA* | intergenic |
|  | rs34304371 | rs34304371 | 7:12147500 | A | 1.58 | 0.33 | 1.32×10^-6^ | 0.194 | 0.084 | *TMEM106B* | intergenic |
|  | rs35571868 | rs35571868 | 10:6440491 | C | -1.16 | 0.25 | 2.96×10^-6^ | 0.732 | 0.866 | *PRKCQ* | intronic |
|  | rs11044926 | rs11044926 | 12:20046132 | G | 5.30 | 1.11 | 1.91×10^-6^ | 0.053 | 0.008 | *PDE3A* | intergenic |
|  | rs141181294 | rs141181294 | 16:87887318 | AGCTTTCTG | 1.60 | 0.33 | 9.50×10^-7^ | 0.202 | 0.088 | *SLC7A5* | intronic |
| Psychosis  (N=26) | rs143606502 | rs143606502; rs199670042 | 2:24206911 | A | 9.55 | 2.07 | 3.91×10^-6^ | 0.115 | 0.011 | *UBXN2A* | intronic |
|  | rs61353631 | rs61353631 | 3:197312702 | A | 11.92 | 2.51 | 2.09×10^-6^ | 0.104 | 0.008 | *BDH1* | intergenic |
|  | rs1250028 | rs1250028 | 9:117857487 | G | -8.10 | 1.76 | 4.19×10^-6^ | 0.869 | 0.972 | *TNC* | intronic |
|  | rs139418206 | rs139418206 | 10:106666885 | CTG | 2.64 | 0.58 | 4.40×10^-6^ | 0.326 | 0.107 | *SORCS3* | intronic |
|  | rs79782108 | rs79782108; rs11847467 | 14:20441298 | A | 11.12 | 2.17 | 2.93×10^-7^ | 0.124 | 0.008 | *OR4K15* | intergenic |
|  | rs183262 | rs183262 | 16:25617424 | G | 5.35 | 1.16 | 4.40×10^-6^ | 0.173 | 0.025 | *HS3ST4* | intergenic |
|  | rs189630170 | rs189630170 | 19:1569748 | T | 7.97 | 1.75 | 4.98×10^-6^ | 0.133 | 0.014 | *MEX3D* | intergenic |
|  | rs12610945 | rs12610945 | 19:45949578 | T | 1.87 | 0.38 | 1.13×10^-6^ | 0.528 | 0.204 | *ERCC1* | intronic |
|  | rs76512765 | rs76512765 | 20:18259316 | T | 2.46 | 0.53 | 3.74×10^-6^ | 0.355 | 0.112 | *ZNF133* | intergenic |
| Seizure or Psychosis  (N=103) | rs141540533 | rs141540533 | 1:94500997 | A | 4.16 | 0.90 | 3.73×10^-6^ | 0.051 | 0.008 | *ABCA4* | intronic |
|  | **rs191085932** | rs191085932 | 4:77499261 | C | 3.10 | 0.64 | 1.41×10^-6^ | 0.068 | 0.011 | *SHROOM3* | intronic |
|  | rs10869726 | rs10869726; rs33951103 | 9:78804704 | A | 1.22 | 0.25 | 1.35×10^-6^ | 0.218 | 0.101 | *PCSK5* | intronic |
|  | rs200489169 | rs200489169 | 10:31774777 | T | 3.37 | 0.71 | 1.98×10^-6^ | 0.066 | 0.014 | *ZEB1* | intronic |
|  | rs188962092 | rs188962092 | 11:13506365 | A | 2.40 | 0.52 | 3.36×10^-6^ | 0.088 | 0.027 | *PTH* | intergenic |
|  | rs5790809 | rs5790809; rs200065590 | 11:30312421 | G | -0.72 | 0.15 | 2.57×10^-6^ | 0.444 | 0.617 | *ARL14EP* | intergenic |
|  | rs117900986 | rs117900986 | 12:109186093 | C | 2.26 | 0.49 | 3.46×10^-6^ | 0.092 | 0.024 | *SSH1* | exonic |
|  | **rs4508395** | rs4508395**;** rs11072705; rs71400903 | 15:78239225 | T | 1.26 | 0.24 | 1.97×10^-7^ | 0.262 | 0.131 | *LINGO1* | intergenic |
|  | **rs6023524** | rs6023524 | 20:53310831 | C | -0.76 | 0.17 | 4.62×10^-6^ | 0.179 | 0.329 | *DOK5* | intergenic |

^*^ Overlapped loci in two analysis set was in bold

^†^ For gene mapping, we used the nearest gene from Open Targets Genetics (genetics.opentargets.org) or intronic gene from Variant Effect Predictor (VEP; https://asia.ensembl.org/info/docs/tools/vep/index.html) or dbSNP (<https://www.ncbi.nlm.nih.gov/snp/>). Variant annotation was performed using the VEP from Open Targets Genetics, and when unavailable, information was obtained from dbSNP.

**Supplementary table 2. Mapping of 25 genes to NPSLE-associated loci**

| **ENSG** | **Gene *** | **Chr** | **Start** | **End** | **Type** | **Independent Significant SNPs** |
| --- | --- | --- | --- | --- | --- | --- |
| ENSG00000156011 | *PSD3* | 8 | 18384811 | 18942240 | protein_coding | rs201681644 |
| ENSG00000148541 | *FAM13C* | 10 | 61005890 | 61122939 | protein_coding | rs75831230 |
| ENSG00000165449 | *SLC16A9* | 10 | 61410523 | 61495760 | protein_coding | rs75831230 |
| ENSG00000108091 | *CCDC6* | 10 | 61548521 | 61666414 | protein_coding | rs75831230 |
| ENSG00000151150 | *ANK3* | 10 | 61786056 | 62493248 | protein_coding | rs75831230 |
| ENSG00000170312 | *CDK1* | 10 | 62538089 | 62554610 | protein_coding | rs75831230 |
| ENSG00000072422 | *RHOBTB1* | 10 | 62629196 | 62761198 | protein_coding | rs75831230 |
| ENSG00000183715 | *OPCML* | 11 | 132284871 | 133402414 | protein_coding | rs56405523 |
| ENSG00000151502 | *VPS26B* | 11 | 134094539 | 134117686 | protein_coding | rs56405523 |
| ENSG00000135116 | *HRK* | 12 | 117293949 | 117319246 | protein_coding | rs551340 |
| ENSG00000088992 | *TESC* | 12 | 117476728 | 117537284 | protein_coding | rs551340 |
| ENSG00000089250 | *NOS1* | 12 | 117645947 | 117889975 | protein_coding | rs551340; rs62838161 |
| ENSG00000140090 | *SLC24A4* | 14 | 92788925 | 92962596 | protein_coding | rs34875253 |
| ENSG00000100599 | *RIN3* | 14 | 92980118 | 93155339 | protein_coding | rs34875253 |
| ENSG00000140368 | *PSTPIP1* | 15 | 77285700 | 77329673 | protein_coding | rs4508395 |
| ENSG00000140391 | *TSPAN3* | 15 | 77336359 | 77376326 | protein_coding | rs4508395 |
| ENSG00000173517 | *PEAK1* | 15 | 77400471 | 77712486 | protein_coding | rs4508395 |
| ENSG00000140382 | *HMG20A* | 15 | 77712754 | 77777949 | protein_coding | rs4508395 |
| ENSG00000169783 | *LINGO1* | 15 | 77905369 | 78113242 | protein_coding | rs4508395 |
| ENSG00000167202 | *TBC1D2B* | 15 | 78276378 | 78370066 | protein_coding | rs4508395 |
| ENSG00000183476 | *SH2D7* | 15 | 78370150 | 78397251 | protein_coding | rs4508395 |
| ENSG00000136425 | *CIB2* | 15 | 78396948 | 78423886 | protein_coding | rs4508395 |
| ENSG00000166411 | *IDH3A* | 15 | 78423840 | 78464291 | protein_coding | rs4508395 |
| ENSG00000103740 | *ACSBG1* | 15 | 78459810 | 78538030 | protein_coding | rs4508395 |
| ENSG00000140403 | *DNAJA4* | 15 | 78556428 | 78574538 | protein_coding | rs4508395 |

^*^ Nine loci significantly associated with NPSLE (P<5×10^-6^) were mapped to 25 genes by FUMA platform

**Supplementary table 3. Genes significantly upregulated in brain regions including basal ganglia, cortex, and hippocampus**

| **Gene Set** | **N genes in set *** | **N overlapped genes** | ***P*** | **adjusted *P*** ^†^ | **Genes** |
| --- | --- | --- | --- | --- | --- |
| Brain_Nucleus_accumbens_basal_ganglia | 1839 | 6 | 6.93×10^-5^ | 3.74×10^-3^ | *PSD3, OPCML, NOS1, SLC24A4, LINGO1, DOK5* |
| Brain_Cortex | 2167 | 6 | 1.77×10^-4^ | 9.53×10^-3^ | *PSD3, ANK3, OPCML, SLC24A4, LINGO1, DOK5* |
| Brain_Putamen_basal_ganglia | 1350 | 5 | 2.22×10^-4^ | 0.012 | *PSD3, OPCML, NOS1, LINGO1, DOK5* |
| Brain_Frontal_Cortex_BA9 | 2429 | 6 | 3.36×10^-4^ | 0.018 | *PSD3, ANK3, OPCML, SLC24A4, LINGO1, DOK5* |
| Brain_Hippocampus | 1546 | 5 | 4.22×10^-4^ | 0.023 | *PSD3, ANK3, OPCML, LINGO1, DOK5* |
| Brain_Caudate_basal_ganglia | 1707 | 5 | 6.71×10^-4^ | 0.036 | *PSD3, OPCML, NOS1, LINGO1, DOK5* |

* Pre-defined gene set in the GTEx v8

^†^ Significant gene sets were represented (adjusted P value (FDR) < 0.05)

**Supplementary table 4. eQTL–gene–brain region pairs of LD SNPs linked to independent SNPs of NPSLE**

| **NPSLE set** | **N lead/ independent SNPs** | **N LD SNPs (R^2^>0.6)** | **Brain regions in the GTEx v8** | | | **PsychENCODE** | | **BRAINEAC** | | |
| --- | --- | --- | --- | --- | --- | --- | --- | --- | --- | --- |
|  |  |  | **N eQTL-gene pair** | **paired genes** | **Detailed brain region** | **N eQTL-gene pair** | **paired genes** | **N eQTL-gene pair** | **paired genes** | **Detailed brain region** |
| **NPSLE** | 9/12 | 150 | 18 | *CIB2*  *LINGO1*  *IDH3A* | Cerebellar Hemisphere | 3 | *TESC*  *CIB2* | 0 | - | - |
|  |  |  | 35 | *DNAJA4*  *CIB2*  *LINGO1*  *IDH3A* | Cerebellum |  |  |  |  |  |
| **Seizure** | 7/7 | 137 | 7 | *THSD7A* | Cerebellum | 23 | *SCIN*  *VWDE* | 0 | - | - |
| **Psychosis** | 9/11 | 348 | 3 | *OPA3* | Cortex | 28 | *KLC3*  *SORCS3* | 2 | *TNRC6A* | Cerebellar cortex |
|  |  |  | 10 | *CD3EAP* | Nerve Tibial |  |  |  |  |  |
| **Seizure or Psychosis** | 9/15 | 508 | 163 | *ARL14EP* | Caudate basal ganglia | 394 | *FSHB*  *ARL14EP*  *MPPED2*  *CIB2* | 168 | *GOLGA6A*  *GOLGA6B*  *GOLGA6C*  *GOLGA6D* | Cerebellar cortex |
|  |  |  | 311 | *ARL14EP*  *LINGO1*  *CIB2*  *IDH3A* | Cerebellar Hemisphere |  |  |  |  |  |
|  |  |  | 598 | *ARL14EP*  *LINGO1*  *CIB2*  *IDH3A*  *DNAJA4* | Cerebellum |  |  |  |  |  |
|  |  |  | 207 | *ARL14EP* | Cortex |  |  |  |  |  |
|  |  |  | 71 | *ARL14EP* | Frontal Cortex BA9 |  |  |  |  |  |
|  |  |  | 69 | *ARL14EP* | Hippocampus |  |  |  |  |  |
|  |  |  | 109 | *ARL14EP*  *FSHB* | Hypothalamus |  |  |  |  |  |
|  |  |  | 114 | *ARL14EP* | Nucleus accumbens basal ganglia |  |  |  |  |  |
|  |  |  | 68 | *ARL14EP* | Putamen basal ganglia |  |  |  |  |  |
|  |  |  | 89 | *ARL14EP* | Spinal cord cervical c-1 |  |  |  |  |  |
|  |  |  | 228 | *ARL14EP*  *MPPED2* | Nerve Tibial |  |  |  |  |  |

**
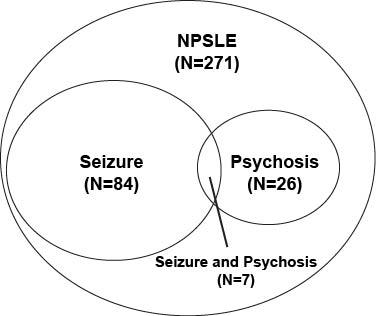
**

**Supplementary figure 1. NPSLE, seizure and psychosis in SLE patients.**


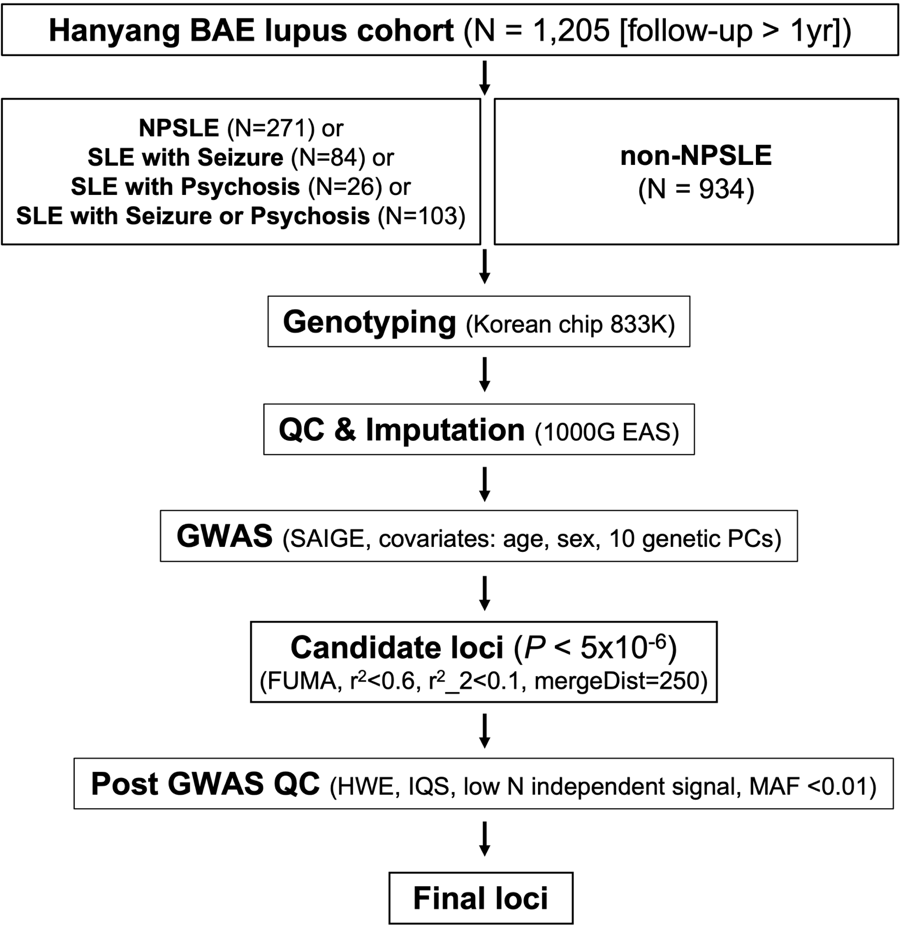


**Supplementary figure 2. Workflow of the genetic analyses for identifying NPSLE-associated loci.**


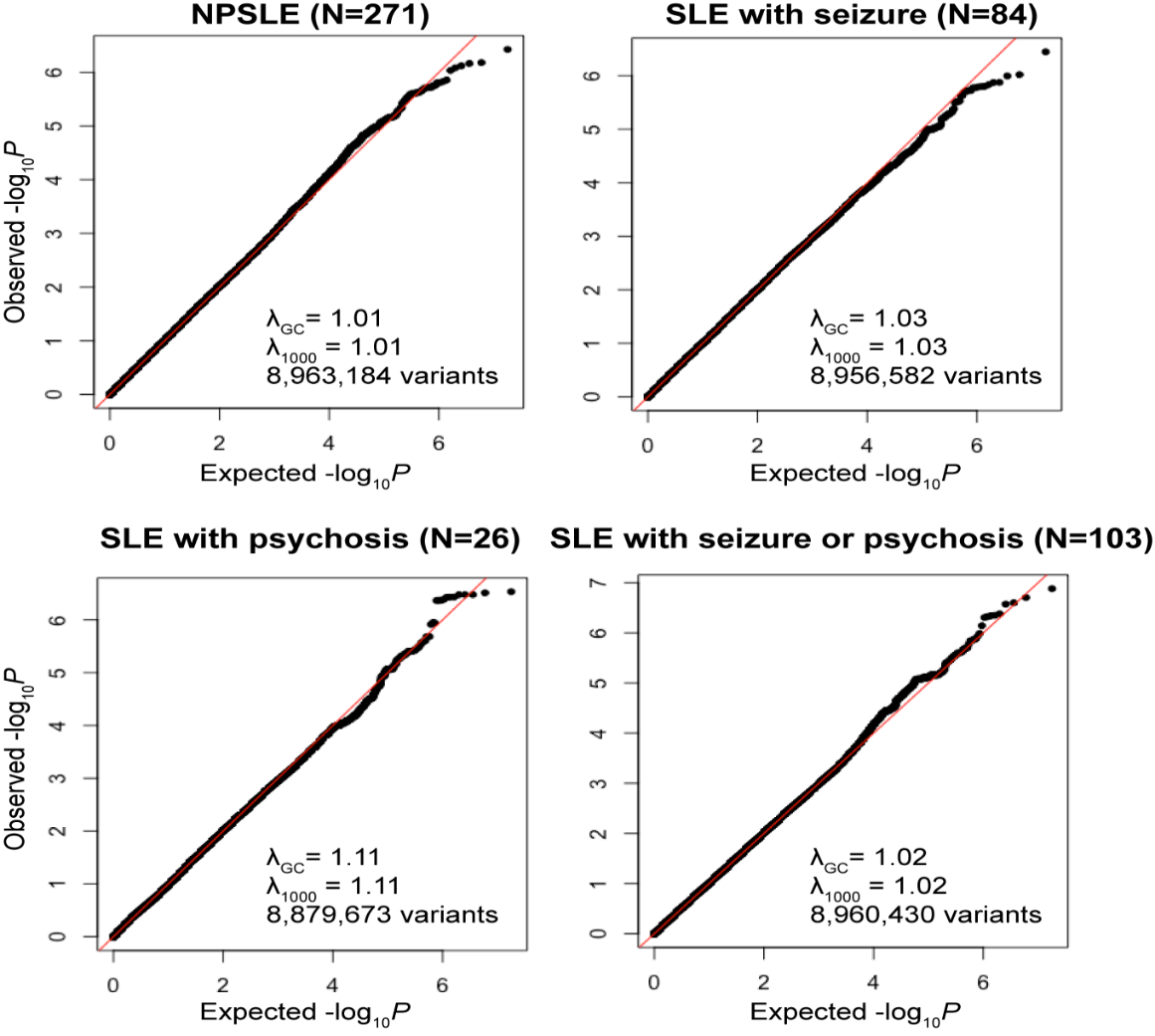


**Supplementary figure 3. Quantile–quantile (QQ) plot of NPSLE, seizure, and/or psychosis set.**

*genomic inflation factor (λ_GC_)

**Supplementary figure 4. Distribution of organ damage across SDI domains between NPSLE and non-NPSLE patients.**

*Musculoskeletal damage was significantly higher in the NPSLE compared with non-NPSLE (OR 1.81, *P*=2.65×10⁻⁴). *P* values were calculated based on logistic regression adjusted for age, sex, disease duration, hypertension, and antiphospholipid antibodies. Premature gonadal failure (n ≤5) was excluded from the analysis. The significance threshold was set using the Bonferroni method, adjusting for 10 SDI domains (P<0.005 [=0.05/10]).

**
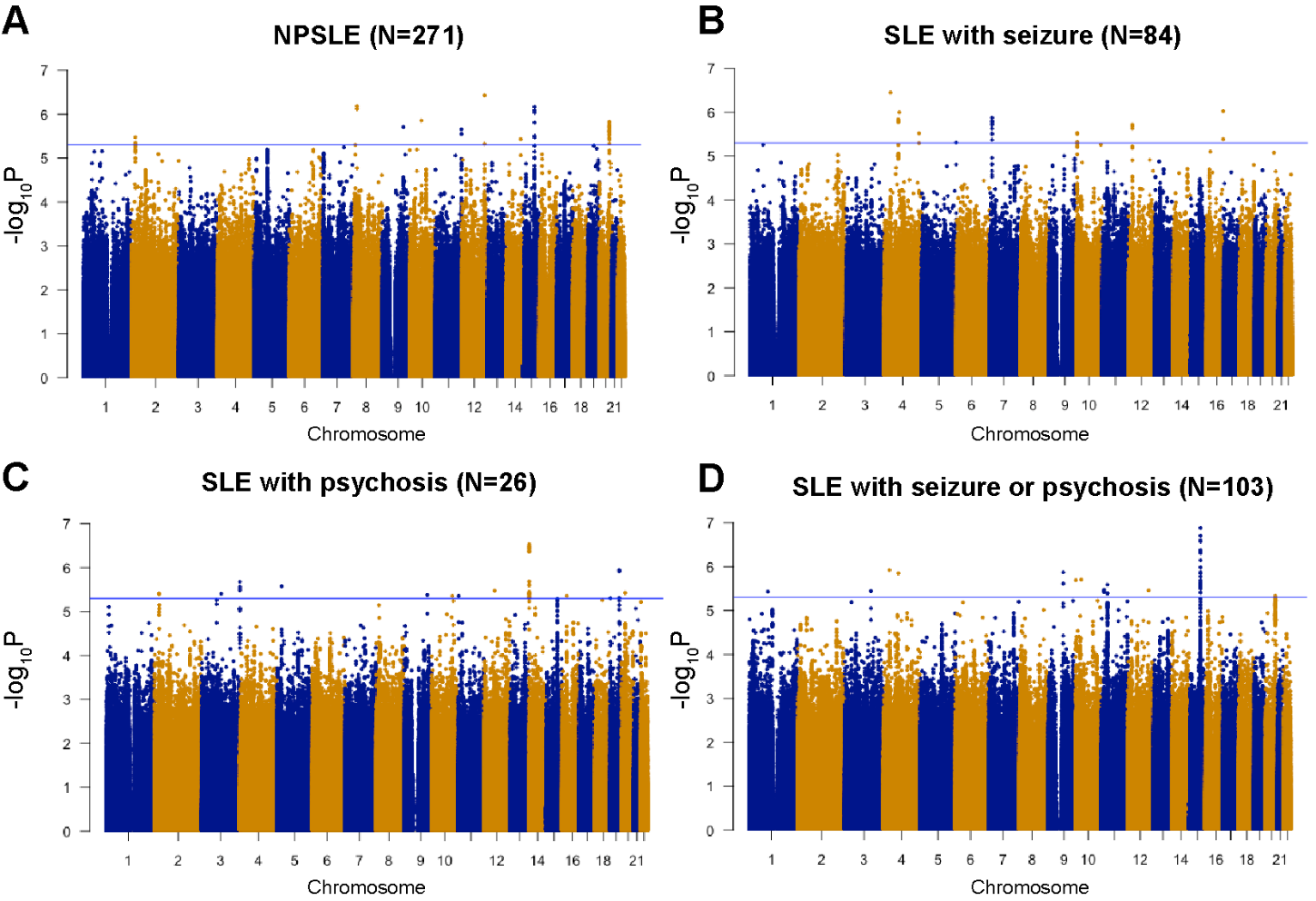
**

**Supplementary figure 5. Genome-wide association analysis of NPSLE, seizure, or psychosis set.**

**
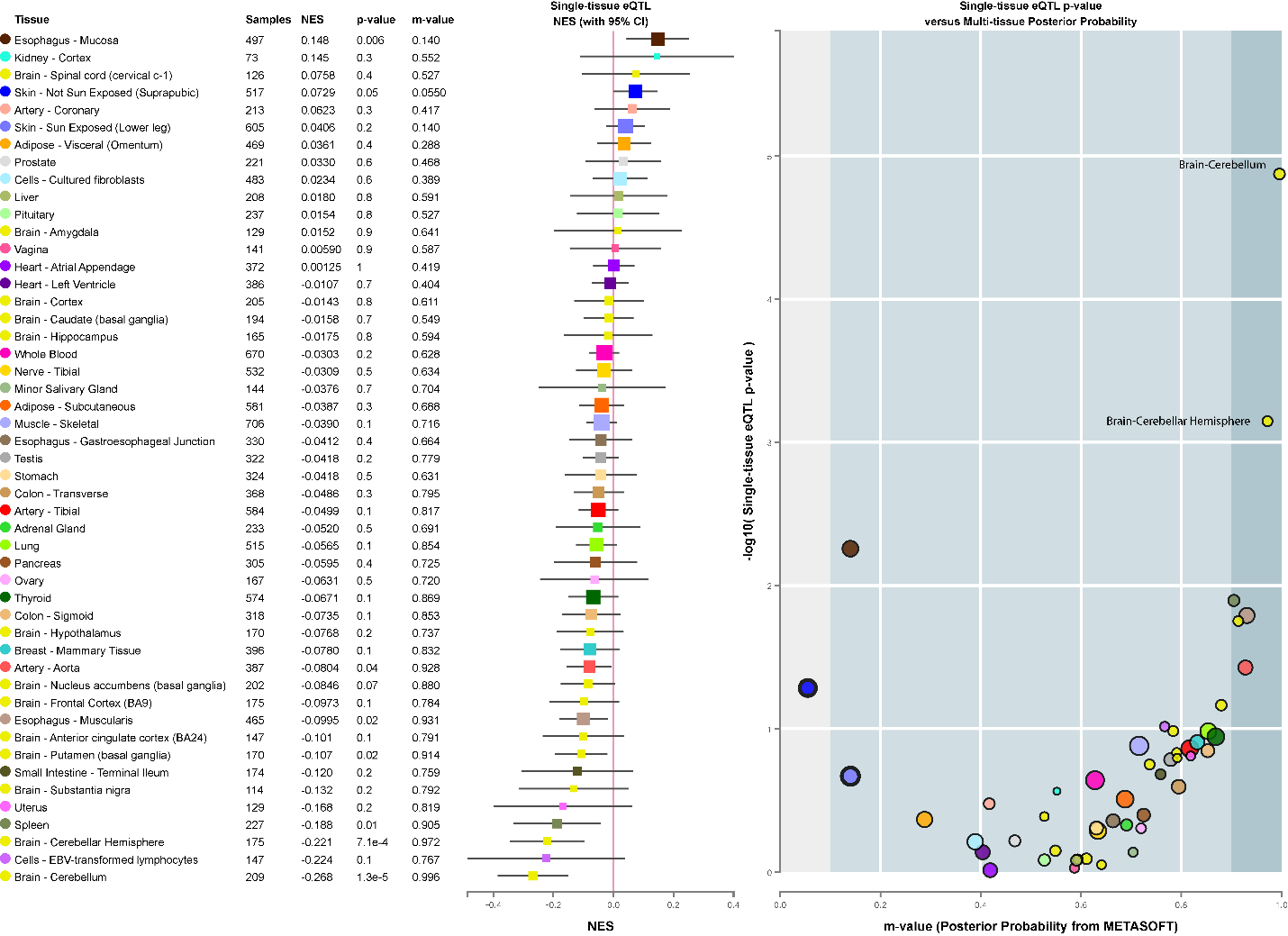
**

**Supplementary figure 6. Multi-tissue eQTL Comparison for rs4508395–DNAJA4 pair.**
